# El CYFRA 21-1 en pacientes con sospecha de cáncer: evaluación de un punto de corte óptimo para evaluar la eficacia diagnóstica y el valor pronóstico

**DOI:** 10.1515/almed-2020-0092

**Published:** 2020-10-30

**Authors:** Sonsoles Garcia-Valdecasas Gayo, Maria Jesus Ruiz-Alvarez, Daniel Gonzalez-Gay, Raquel Ramos-Corral, Eva Marquez-Lietor, Nazaret Del Amo, Maria del Carmen Plata, Raquel Guillén-Santos, Ignacio Arribas, Fernando Cava-Valenciano

**Affiliations:** Departamento de Análisis Clínicos, BR Salud, Hospital Infanta Sofía, Madrid, España; Departamento de Análisis Clínicos, Hospital Universitario Ramón y Cajal University, Madrid, España

**Keywords:** cáncer de pulmón, cáncer de pulmón no microcítico (CPNM), cáncer de vejiga, CYFRA 21-1, marcador tumoral, pronóstico, punto de corte

## Abstract

**Objetivos:**

El punto de corte utilizado del CYFRA 21-1 como marcador tumoral influye considerablemente en su utilidad diagnóstica y pronóstica. El objetivo de este estudio es establecer un punto de corte óptimo de CYFRA 21-1 que tenga validez diagnóstica, determinado con la tecnología Lumipulse^®^ en pacientes con sospecha de cáncer. El objetivo secundario de este estudio es determinar si los niveles de CYFRA 21-1 tienen valor pronóstico.

**Métodos:**

Se llevó a cabo un estudio retrospectivo en una muestra compuesta por 284 pacientes con sospecha de enfermedad maligna procedentes de seis hospitales de Madrid. El punto de corte se obtuvo mediante la curva ROC y el test de Youden. La validez diagnóstica se evaluó de acuerdo con la sensibilidad, especificidad, valores predictivos y ratios de probabilidad. El valor pronóstico de CYFRA 21-1 se determinó por medio de la regresión logística múltiple. Se confirmaron un total de 32 casos de cáncer.

**Resultados:**

El punto de corte más óptimo fue 3,15 ng/mL. Este punto de corte mostró mejor especifidad, de 93,63% (89,66–96,16; IC 95%), Valor Predictivo Positivo (44,54–75,38; IC 95%) y Razón de Probabilidad Positiva 12,65 (7,64–20,95; IC 95%) que el punto de corte recomendado por Fujirebio^®^ (1,8 ng/mL); con una especifidad de 73,71% (67,72–78,95; IC 95%), Valor Predictivo Positivo: 29,79% (21,02–40,23; IC 95%) y una Razón de Probabilidad Positiva de 3,43 (2,71–4,35; IC 95%), mejorando la precisión diagnóstica actual. En el análisis multivariante, se confirmó que los niveles elevados de CYFRA 21-1 (>3,15 ng/mL) son un factor pronóstico desfavorable.

**Conclusiones:**

El mejor punto de corte obtenido para CYFRA 21-1 fue de 3,15 ng/mL en pacientes con sospecha de cáncer. Este nuevo punto de corte reduce la tasa de falsos positivos y mejora la eficacia diagnóstica de CYFRA 21-1 como marcador tumoral, así como su asociación con la mortalidad.

## Introductión

El antígeno del fragmento de citoqueratina 19 (CYFRA 21-1) pertenece a la familia de las citoqueratinas y forman los filamentos del citoesqueleto de las células epiteliales [1]. El CYFRA 21-1 es útil como marcador tumoral, especialmente en el cáncer de pulmón no microcítico (CPNM), junto con el antígeno carcinoembrionario (CEA) y el antígeno asociado al carcinoma de células escamosas (CCE) [2], [3], pero también para otros tipos de tumores epiteliales como el cáncer de vejiga [4]. Esta elevación en los tumores se puede deber a la lisis celular, a través de la cual se libera material del interior de la célula al torrente sanguíneo, incluido el CYFRA 21-1, mediante la acción de las proteasas que degradan los filamentos de citoqueratina [5].

Tras la enfermedad coronaria, el cáncer es la segunda causa de muerte en el mundo, siendo el cáncer de pulmón el más mortal de todos los tipos de cáncer [6]. El diagnóstico temprano del cáncer pulmonar es fundamental y, aunque los avances en las técnicas de imagen han permitido adelantar su diagnóstico, la mayor parte de los pacientes con CPNM se encuentran ya en un estadio avanzado de la enfermedad, para el cual existe un número limitado de opciones terapéuticas [7]. La detección temprana de progresión de la enfermedad es también crucial, ya que con ella se ahorra tiempo y costes y se evitan los efectos secundarios innecesarios provocados por la exposición del paciente a tratamiento ineficaces [8]. Los marcadores tumorales séricos se pueden utilizar de forma complementaria en la detección del cáncer, la progresión tumoral y la monitorización del tratamiento [9], [10], [11]. De hecho, los marcadores tumorales suelen ser más útiles por su valor pronóstico y, aunque su valor diagnóstico es bajo, en la práctica clínica, los médicos suelen solicitar la medición de marcadores tumorales para tal fin, como complemento a las pruebas clínicas y otras pruebas diagnósticas. La eficacia diagnóstica y pronóstica de los marcadores tumorales depende en gran medida del punto de corte establecido por el laboratorio y determina su especifidad y sensibilidad y, por tanto, su efectividad [12]. Sin embargo, el punto de corte de CYFRA 21-1 no está claro y no se ha estandarizado entre los diferentes métodos analíticos [13]. Aún no se ha establecido un punto de corte óptimo estimado con tecnología (Lumipulse^®^ Inc). Además, para establecer un punto de corte óptimo, se debe tener en cuenta que también se observan niveles elevados de CYFRA 21-1 en otras enfermedades no oncológicas, como la insuficiencia renal, la enfermedad hepática y las enfermedades pulmonares obstructivas (como la enfermedad pulmonar obstructiva crónica, las infecciones, etc), lo que dificulta el diagnóstico del cáncer.

El punto de corte de CYFRA 21-1 medido con un analizador Lumipulse^®^ recomendado por el fabricante es de 1,8 ng/mL (Fujirebio^®^ Inc). Este punto de corte se determinó a partir de datos obtenidos a partir de adultos sanos. No obstante, en la práctica clínica, se ha demostrado que este punto de corte es demasiado bajo para poder emplearlo en pacientes con sospecha de cáncer, ya que la mayoría de estos pacientes tienen niveles elevados de CYFRA 21-1 aun cuando presentan una enfermedad benigna. Además, estos valores confunden a los facultativos, y por lo tanto en este caso es más útil aumentar la especifidad de la prueba diagnóstica. De este modo, hemos realizado este estudio para determinar el punto de corte del CYFRA 21-1 determinado mediante tecnología Lumipulse^®^ para mejorar la eficacia diagnóstica del marcador y evaluar la información pronóstica.

## Materiales y métodos

Se realizó un estudio observacional retrospectivo de los niveles séricos de CYFRA 21-1 en 284 pacientes consecutivos con sospecha de enfermedad maligna atendidos entre enero y marzo de 2018, siete de los cuales eran hombres (55%) y 127 eran mujeres (45%), todos de entre 25 y 94 años de edad (media: 68 años)

El criterio de inclusión fue pacientes de más de 18 años con sospecha de cáncer. La sospecha de cáncer se determinó en base a criterios clínicos como la fatiga, el síndrome constitucional, tos persistente, dolor localizado y disnea. El diagnóstico definitivo de cáncer se confirmó mediante un análisis histológico de tejido tumoral, que es la técnica estándar utilizada. La presencia de enfermedad renal se determinó mediante valores de filtrado renal (fórmula CKD-EPI) inferiores a 60 mL/min/1,73 m^2^ y la enfermedad hepática mediante valores de bilirrubina total superiores a 4 mg/dL.

Los tipos histológicos de cáncer se clasificaron de acuerdo con las recomendaciones de la Organización Mundial de la Salud [14] para los tumores pulmonares [14] y el cáncer de vejiga [15].

Además, se realizó un seguimiento de los mismos 284 pacientes durante 12 meses, revisando sus historias clínicas codificadas, para identificar la relación entre los niveles séricos de CYFRA 21-1 y el resultado clínico (muerte) para determinar su valor pronóstico.

Las muestras de sangre se recogieron en cada hospital, se centrifugaron a 3500 g durante 10 min, y el suero se conservó a −40 °C.

Los niveles séricos de CYFRA 21-1 se midieron mediante inmunoensayo enzimático quimioluminiscente (CLEIA) en un analizador automático Lumipulse^®^ (Fujirebio^®^ Inc, Japan) siguiendo las instrucciones del fabricante. Tras la medición cuantitativa del índice hemolítico en todas las muestras, ninguna estaba hemolizada.

El ensayo fue lineal dentro del intervalo 0,5–100 ng/mL. El límite inferior de detección fue 0,32 ng/mL y la imprecisión fue inferior a 4,2% según el protocolo EP5-A2 [16] del Clinical and Laboratory Standards Institute (CLSI), quien recomienda una variabilidad deseable de menos de 5% [17].

La confidencialidad de los datos de los pacientes se garantizó en todo momento mediante la codificación de los números de historia médica y revisando sus historias clínicas de forma anónima. Además, los pacientes dieron su consentimiento verbal a la extracción de la muestra y el análisis de CYFRA 21-1 para evaluar el valor diagnóstico y pronóstico de la prueba, extracción que se realizó coincidiendo con las extracciones rutinarias, con el fin de no realizar una extracción adicional de sangre. El proyecto fue autorizado por el Comité de Ética local. Este estudio cumple con la Declaración de Helsinki.

-Análisis estadístico-

Los niveles séricos de CYFRA 21-1 están expresados como mediana y rango intercuartílico (IQR).

El punto de corte óptimo de CYFRA 21-1 se obtuvo a través de la curva ROC y el test de Youden. La eficacia diagnóstica se basó en la sensibilidad, especifidad, valores predictivos negativo y positivo (VPP, VPN), las ratios de probabilidad (PLR, NLR) y el análisis de la característica operativa del receptor (curvas ROC) con el área bajo la curva (AUC).

En el estudio pronóstico, se ha evaluado la asociación entre cada variable del estudio y la variable de resultado (mortalidad) para lo cual se codificaron las variables cuantitativas como dicotómicas con respecto a la mediana. La presencia e interacción de factores de confusión se analizó mediante análisis estratificado. Finalmente, se construyó un modelo de regresión logística múltiple para identificar las variables independientemente asociadas con mortalidad. La razón de probabilidades (OR) se calculó con IC al 95%. La prueba de Hosmer–Lemeshow se empleó para comprobar la bondad de ajuste del modelo.

Las estadísticas están expresadas con intervalos de confianza al 95% (IC 95%) y se realizaron con el programa SPSS (versión 11,0; Chicago, USA). Este estudio se realizó de conformidad con la declaración STARD [18] y las recomendaciones REMARK [19].

## Resultados

Tras revisar todas las historias clínicas, se obtuvieron un total de 32 diagnósticos definitivos de enfermedad oncológica confirmados mediante análisis histológico de biopsia (11%), mientras que 252 (89%) tuvieron otros diagnósticos. De un total de 252 pacientes en el grupo no oncológico (otros diagnósticos), 31 pacientes recibieron un diagnóstico de enfermedad hepática; 26 de insuficiencia renal; 21 de enfermedad pulmonar obstructiva crónica (COPD). En la Tabla 1 se muestran los diferentes diagnósticos.

**Tabla 1:** Descripción del tipo de enfermedad oncológica o enfermedad no oncológica y concentración de CYFRA 21.1 (ng/mL).

Enfermedad oncológica	n	Enfermedad no oncológica	n
**Cáncer pulmonar**		**Hepatopatía**	31
Adenocarcinoma	9	**Insuficiencia renal**	26
Carcinoma de células escamosas	6	**Enfermedad pulmonar obstructiva crónica (EPOC)**	21
Tumor carcinoide	2	**Infección urinaria**	10
Carcinoma mucoepidermoide	1	**Infección respiratoria**	9
Carcinoma de células no microcíticas (sin especificar)	6	**Fallo cardíaco**	9
**Cáncer de vejiga**		**Gastritis**	5
Adenocarcinoma	3	**Enfisema**	4
**Cáncer metastásico**		**Otras enfermedades benignas**	137
Cáncer de mama metastásico	1	(anemia por deficiencia de hierro, disnea, colecistitis, sin enfermedad, diarrea, ansiedad, dolor de cabeza, pólipos y síndrome constitucional)	
Cáncer de colon metastásico	1			
Cáncer gástrico mestastásico	1			
Melanoma metastásico	1			
Tumor metastásico (origen sin especificar)	1			
**Total, (n)**	**32**		**252**
CYFRA 21-1, mediana (IQR)	4,75 (3,89–7,70)	CYFRA 21-1, mediana (IQR)	1,20 (1,10–1,30)

Concentraciones de CYFRA 21-1: mediana (ng/mL) e IQR (rango intercuartílico).

En el grupo de enfermedad oncológica, la mayoría de los tumores diagnosticados corresponden a cáncer pulmonar (75%), el 10% a cáncer urotelial y el resto (15%) a tumores metastásicos, tal como se muestra en la Tabla 1. Del total de diagnósticos oncológicos, la mayoría de las histologías correspondieron a adenocarcinomas. La mediana de CYFRA 21-1 fue significativamente superior en el grupo de enfermedad oncológica: 4,75 (3,89–7,70), frente al de enfermedad no oncológica (otros diagnósticos): 1,20 (1,10–1,30). Estos resultados se han obtenido mediante el test de Mann-Whitney U (p<0,05), dado que los resultados seguían una distribución no paramétrica.

Cuando empleamos el punto de corte actual (1,8 ng/mL) recomendado por Fujirebio^®^, observamos un elevado índice de falsos positivos (66 falsos positivos: 23%) frente a 16 verdaderos positivos (5,7%), de modo que el análisis estadístico mostró una baja especifidad, valor predictivo positivo (VPP) y ratio de probabilidad positiva (RPP) (Tabla 2).

**Tabla 2:** Exactitud del Cyfra 21.1 en función de distintos puntos de corte.

	Valor límite 1.8 ng/mL (IC 95%)	Valor límite 3.15 ng/mL (IC 95%)	Valor límite 3.15 ng/mL sin interferencia^a^ (IC 95%)
Sensibilidad	90.32%	80.65%	79.31%
(IC 95%)	(73,10–97,47)	(61,94–91,88)	(60,73–90,15)
Especifidad	73.71%	93.63%	95.54%
(IC 95%)	(67,72–78,95)	(89,66–96,16)	(92,12–97,35)
Valor predictivo positivo	29.79%	60.98%	69.70%
(IC 95%)	(21,02–40,23)	(44,54–75,38)	(52,98–80,32)
Valor predictivo negativo	98.40%	97.51%	97.27%
(IC 95%)	(95,03–99,59)	(94,40–98,98)	(93,87–98,11)
Razón de probabilidad positiva	3.43	12.65	17.77
(IC 95%)	(2,71–4,35)	(7,64–20,95)	(11,23–24,75)
Razón de probabilidad negativa	0.13	0.21	0.22
(IC 95%)	(0,04–0,39)	(0,10–0,42)	(0,11–0,43)
Prueba de Youden	65.50%	71.80%	73.90%

^a^Sin interferencia: sin pacientes con enfermedad renal y hepática. Enfermedad renal definida como valores de filtrado renal de (CKD-EPI) <60 mL/min/1,73 m^2^ o creatinina sérica <1,3 mg/dL y enfermedad hepática definida como bilirrubina total >4 mg/dL,Los resultados se expresan en porcentajes (%) y los intervales se expresan en IC 95%. Intervalo de confianza.

**Figura 1:**
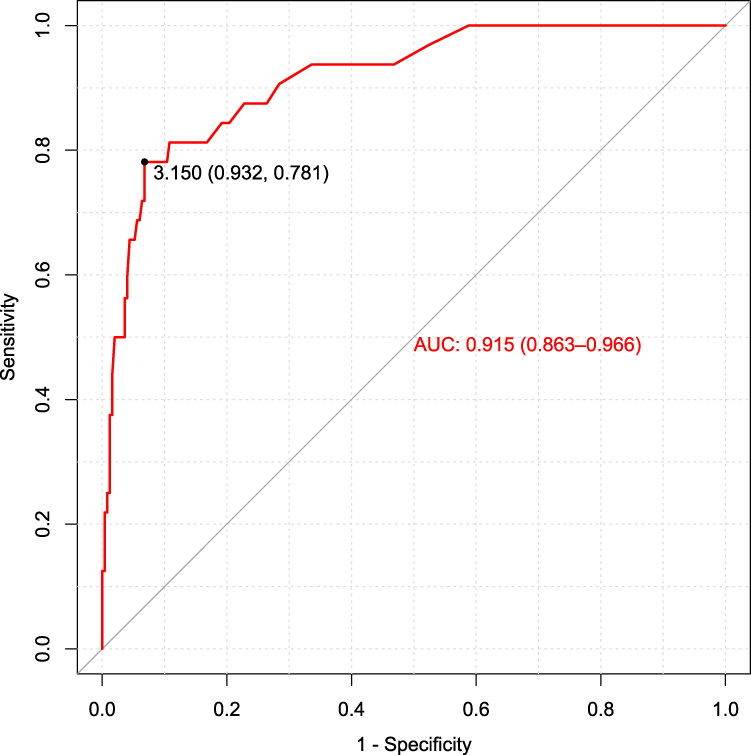
Representación gráfica de las diferentes eficiencias diagnósticas (sensibilidad frente a especifidad) según los diferentes valores límite.

Por lo tanto, nos propusimos determinar el mejor punto de corte de CYFRA 21-1 en nuestra población mediante el test de Youden y la curva ROC (Figura 1), que mostró que 3,15 ng/mL era el punto de corte óptimo (71,8% en la prueba de Youden) con una AUC de 0,915 (0,863–0,966; IC 95%). Este punto de corte mostró tener una mejor especifidad: 93,63% (89,66–96,16; IC 95%), VPP: 60,98% (44,54–75,38; IC 95%) y una RPP de 12,65 (7,64–20,95; IC 95%), que el punto de corte actualmente recomendado por *Fujirebio* ^®^ *Inc* (1,8 ng/mL): Especifidad: 73,71% (67,72–78,95; IC 95%), VPP: 29,79% (21,02–40,23; IC 95%) y una RPP de 3,43 (2,71–4,35; IC 95%), mejorando la precisión diagnóstica de CYFRA 21-1 como marcador tumoral.

Si se hubiera aplicado como criterio de exclusión a aquellos pacientes con enfermedad renal o hepática (presencia de enfermedad renal definida por valores de filtrado renal calculados mediante la fórmula CKD-EPI inferiores a 60 mL/min/1,73 m^2^, y la enfermedad hepática determinada por valores de bilirrubina total superiores a 4 mg/dL), se habría excluido a 29 pacientes de estos grupos, habiéndose obtenido mejores resultados, especialmente en lo relativo a la especifidad, el VPP y la RPP (Tabla 2).

En el estudio pronóstico, durante el seguimiento de 12 meses, se perdió a dos pacientes del grupo de enfermedades benignas, por lo que finalmente se incluyeron un total de 282 pacientes en esta parte del estudio. Se produjeron un total de 32 muertes durante el periodo de seguimiento de 12 meses, a seis de los cuales se les había diagnosticado una enfermedad no oncológica (19%). Del total de 32 muertes, 20 (62%) presentaron elevados niveles de CYFRA 21-1 (1,8 ng/mL cut-off), mientras que 12 pacientes (40%) tenían niveles bajos de CYFRA 21-1.

El análisis de regresión multivariante mostró que el CYFRA 21-1 y la edad estaban independientemente relacionados con la mortalidad (Tabla 3). La prueba de Hosmer–Lemeshow no fue significativa (p = 0,98) a la hora de validar el modelo multivariante.

**Tabla 3:** Modelo final de Regresión Logística multivariante.

Variable	p	OR_ajustado_	IC95%
Edad	0.039	2.67	(1,55–9,12)
CYFRA 21-1	0	1.28	(1,11–1,75)

Intervalo de confianza al 95%. OR, razón de probabilidades.

## Discusión

A menudo los síntomas de cáncer de pulmón no son específicos, como la fatiga, disnea, dolor o tos persistente, especialmente en las etapas más tempranas, ya que se éstos también se manifiestan en otras enfermedades. De este modo, es necesario incluir, además de la historia clínica y los síntomas, un gran número de pruebas para alcanzar el diagnóstico correcto, tales como pruebas analíticas, radiografías, resonancias magnéticas, tomografías computerizadas (TAC) [20], [21], broncoscopia, biopsia, estudios de función pulmonar y, finalmente, una toracocentesis [22], [23]. Las técnicas de imagen tienen una utilidad incuestionable en el diagnóstico del cáncer y a la hora de monitorizar la respuesta al tratamiento, aunque todas ellas tienen un elevado coste y no estás exentas de causar algún daño a los pacientes [24]. El TAC tiene una elevada sensibilidad para la detección de nódulos pulmonares, aunque el paciente recibe una alta dosis de radiación y la mayoría de los nódulos no son carcinogénicos. En un estudio publicado por Swensen et al. en 2005 [25], se detectaron nódulos en más del 70% de los pacientes, mientras que, finalmente, solo el 4% tenían cáncer de pulmón, habiéndose sometido a una elevada dosis de radiación. Por lo tanto, el método ideal debe ser rápido, de bajo coste y tener un efecto mínimo en el paciente. Los biomarcadores tumorales séricos se postulan como una herramienta prometedora, ya que, aunque no tienen una sensibilidad alta, tienen una buena especifidad, son más baratos y solo requieren una muestra de sangre. Además, estos biomarcadores mejorarían las capacidades diagnósticas de los estudios de imagen [26]. No obstante, no existe un criterio uniforme y normalizar algunos biomarcadores tumorales resulta complicado [27]. Además, sus niveles son también más elevados en los pacientes con lesiones benignas, aunque en estos casos, el aumento no es tan pronunciado [28]. Tal es el caso del CYFRA 21-1, que está presente en las células epiteliales sanas y en las células malignas derivadas del epitelio. Por dicha razón, es muy importante establecer un punto de corte adecuado para ayudar a diferenciar un diagnóstico y pronóstico efectivo temprano del cáncer, de las enfermedades benignas. En este estudio, el punto de corte óptimo obtenido con la tecnología *Lumipulse^®^* en nuestra población fue de 3,15 ng/mL, un valor similar a otros establecidos por otros autores para el diagnóstico del cáncer de pulmón, aunque nuestra población no es sana e incluye pacientes con sospecha de cáncer (pacientes sanos y no sanos).

Según la literatura, Rui Huan Xu et al. [29] y Shuwei et al. [30] emplearon una curva ROC para evaluar el punto de corte CYFRA 21-1, estableciendo un punto de corte de 3,95 ng/mL y 3,20 ng/mL, respectivamente, que concuerdan con los datos publicados en la literatura. Liu et al. [31] estableció un punto de corte de 3,3 ng/mL. Trapé et al. [32] realizó un estudio de marcadores tumorales en pacientes con síntomas de cáncer y concluyeron que los marcadores tumorales mejoraban su sensibilidad en el diagnóstico del cáncer empleando diferentes valores límite. Existen numerosos autores que respaldan la combinación de varios marcadores tumorales para mejorar la eficacia diagnóstica en el cáncer de pulmón, como la combinación de CYFRA 21-1 con CEA [33]. Incluso algunos autores sugieren una mayor utilidad diagnóstica al combinar un número mayor de marcadores. Tal es la postura de Molina et al. [34], que concluyeron que la eficacia para diagnosticar el cáncer de pulmón resultó mejor cuando se combinaron 6 marcadores tumorales frente al empleo de esos mismos marcadores de forma individual. Liu et al. [35] recomendaron combinar los mismos seis marcadores tumorales para distinguir los tipos histológicos de cáncer pulmonar.

Las razones de probabilidad, que no se ven afectadas por la prevalencia de la enfermedad, son útiles a la hora de evaluar la probabilidad de que cada paciente padezca la enfermedad tras la determinación del test (probabilidad post-test). Si aplicamos el nomograma de Fagan [36] con nuestra prevalencia (11%) y usamos el punto de corte de 1,8 ng/mL, la probabilidad post-test de padecer cáncer es del 25% (PLR: 3,43) y la probabilidad de descartar el cáncer se reduce a 0,8% (empleando la NLR: 0,13). Sin embargo, si empleamos el punto de corte de 3,15 ng/mL, la probabilidad post-test de padecer cáncer asciende al 65% (PLR: 12,65) y la probabilidad de descartar el cáncer se reduce a 1,8% (empleando la NLR: 0,21).

Dada la considerable interferencia de la enfermedad renal y hepática en los niveles de CYFRA 21-1, recomendamos añadir una nota junto al valor de CYFRA 21-1 en el informe de la analítica que indique "el punto de corte de CYFRA 21-1 recomendado en pacientes con un filtrado renal inferior a 60 mL/min/1,73 m^2^ (o niveles de creatinina superiores a 1,3 mg/dL) es mayor en estos pacientes, por lo que los niveles de CYFRA 21-1 no son interpretables". Del mismo modo, ante la presencia de enfermedad hepática, recomendamos añadir "el punto de corte de CYFRA 21-1 recomendado en pacientes con enfermedad hepática es superior en pacientes con valores de bilirrubina total superiores a 4 mg/dL, por lo que los niveles de CYFRA 21-1 no son interpretables”.

En el análisis multivariante, se confirmó que los niveles elevados de Cyfra 21-1 (>3,15 ng/mL) son un factor pronóstico desfavorable. En un meta-análisis publicado por Holdenvieder et al. [37] se mostró que existe un elevado nivel de evidencia sobre la utilidad clínica de CEA y CYFRA 21-1 para predecir la respuesta al tratamiento en el CPNM. Kulpa et al. [2] también concluyó que el CYFRA 21-1 tiene un elevado valor pronóstico en las etapas tempranas del cáncer de pulmón. En un meta-análisis publicado en 2017 [38] se mostró que los niveles de CYFRA 21-1 indican un estadio avanzado de CPNM. Según Zhang et al. [39], CYFRA 21-1 empleado individualmente tiene mayor valor pronóstico para el cáncer de pulmón que el CEA.

En conclusión, el mejor punto de corte del CYFRA 21-1 (utilizando la tecnología Lumipulse*^®^*) obtenido en nuestra población con sospecha clínica de cáncer fue de 3,15 ng/mL. Este valor es superior que el punto de corte aportado por Fujirebio^®^ (1,8 ng/mL) y aumenta la razón de probabilidad positiva, reduciendo el número de falsos positivos y, por lo tanto, evitando costes innecesarios y molestias al paciente. Este estudio también demuestra que niveles séricos elevados de CYFRA 21-1 empleando el nuevo punto de corte (3,15 ng/mL) pueden ser un marcador no invasivo útil a la hora de identificar el riesgo de muerte temprana causada por CPNM.

### Limitaciones

La prevalencia del cáncer en la población española fue del 1,6% en 2017 (datos oficiales aportados por la Oficina Nacional de Estadística). Sin embargo, la prevalencia en este estudio fue superior (11%), ya que el análisis de marcadores tumorales solo se solicitó en pacientes con sospecha clínica de cáncer, por lo que se sobreestiman los valores predictivos. Sin embargo, la eficacia diagnóstica se debe testar con las razones de probabilidad, que no se ven afectadas por la prevalencia de la enfermedad. Los resultados de este estudio solo se pueden extrapolar a pacientes con sospecha de cáncer.

El punto de corte actual (1,8 ng/mL) deriva en la realización innecesaria de pruebas diagnósticas invasivas, con un aumento de costes y molestias al paciente en forma de efectos secundarios y exposición a radiación.

En este estudio, no hemos tenido en cuenta la influencia del tabaco, sexo o raza en los niveles séricos de CYFRA 21-1. No obstante, algunos estudios en los que se tiene en cuenta la influencia de estos factores no se han observado cambios significativos en los niveles de CYFRA 21-1 [40].

Debido a la significativa influencia de la enfermedad renal, hepática y enfermedad pulmonar benigna en los niveles de CYFRA 21-1, sería interesante estudiar el punto de corte adecuado para establecer el valor diagnóstico y pronóstico en pacientes con enfermedades renales, hepáticas y enfermedades pulmonares benignas empleando la tecnología Lumipulse*^®^.* En este estudio no se pudo realizar, dado que el tamaño de la muestra era insuficiente para establecer un punto de corte, lo que puede ser una línea de investigación interesante en el futuro.
